# A study on feature selection using multi-domain feature extraction for automated k-complex detection

**DOI:** 10.3389/fnins.2023.1224784

**Published:** 2023-09-08

**Authors:** Yabing Li, Xinglong Dong, Kun Song, Xiangyun Bai, Hongye Li, Fakhreddine Karray

**Affiliations:** ^1^School of Computer Science and Technology, Xi’an University of Posts and Telecommunications, Xi’an, Shaanxi, China; ^2^Shaanxi Key Laboratory of Network Data Analysis and Intelligent Processing, Xi’an, Shaanxi, China; ^3^Xi’an Key Laboratory of Big Data and Intelligent Computing, Xi'an, Shaanxi, China; ^4^Machine Learning Department, Mohamed bin Zayed University of Artificial Intelligence, Abu Dhabi, United Arab Emirates

**Keywords:** k-complex, electroencephalography (EEG), multi-domain features, feature selection, detection

## Abstract

**Background:**

K-complex detection plays a significant role in the field of sleep research. However, manual annotation for electroencephalography (EEG) recordings by visual inspection from experts is time-consuming and subjective. Therefore, there is a necessity to implement automatic detection methods based on classical machine learning algorithms. However, due to the complexity of EEG signal, current feature extraction methods always produce low relevance to k-complex detection, which leads to a great performance loss for the detection. Hence, finding compact yet effective integrated feature vectors becomes a crucially core task in k-complex detection.

**Method:**

In this paper, we first extract multi-domain features based on time, spectral analysis, and chaotic theory. Those features are extracted from a 0.5-s EEG segment, which is obtained using the sliding window technique. As a result, a vector containing twenty-two features is obtained to represent each segment. Next, we explore several feature selection methods and compare their performance in detecting k-complex. Based on the analysis of the selected features, we identify compact features which are fewer than twenty-two features and deemed as relevant and proceeded to the next step. Additionally, three classical classifiers are employed to evaluate the performance of the feature selection models.

**Results:**

The results demonstrate that combining different features significantly improved the k-complex detection performance. The best performance is achieved by applying the feature selection method, which results in an accuracy of 93.03% ± 7.34, sensitivity of 93.81% ± 5.62%, and specificity 94.13 ± 5.81, respectively, using a smaller number of the combined feature sets.

**Conclusion:**

The proposed method in this study can serve as an efficient tool for the automatic detection of k-complex, which is useful for neurologists or doctors in the diagnosis of sleep research.

## 1. Introduction

Besides diverse psychophysiological monitoring and medical prevention, sleep estimation hinges on EEG can also play a critical role in the monitoring of sleep disorder disease (Li et al., 2017; Shi et al., 2021). Generally, sleep of human can be split into six stages including wake (stage 0), light stages (stages 1 and 2), delta or deep stages (stages 3 and 4), and rapid eye movement (REM) (Zorick and Smith, 2016). k-complex, one of the hallmarks of stage 2, is a crucially important waveform for sleep analysis (Yücelbaş et al., 2018; Horie et al., 2022). Pursuant to the American Academy of Sleep Medicine (AASM), k-complex is defined as “a well-delineated negative sharp component immediately followed by a positive wave with a duration larger than 0.5 s” (Berry et al., 2015). In community, the most common strategy of detecting k-complex is the expert annotation-based detection of k-complex in EEG signals, which is also considered as the gold standard. Therefore, how to effectively detect k-complex is a challenge. However, the golden standard method is a tedious, time-consuming and expensive task, because it needs the expertise of the clinicians (AL-Salman et al., 2018; Khasawneh et al., 2022). Consequently, a large number of methods for k-complex have been proposed to alleviate the burden of neurologists (Hassan and Bhuiyan, 2017; Dumitrescu et al., 2021; Zhang et al., 2022).

Among these methods, the entire procedure typically consists of three parts: feature extraction, feature selection, and classification. The waveforms of EEG signals during sleep stages include sleep spindles, spikes, vertex sharp waves, alpha bursts, and so on. Because the amplitude of those waveforms varies significantly, it is challenging to detect k-complex using some features directly. Hence, one major issue in the detection system is extracting proper features that effectively discriminate between k-complex and non-k-complex signals. Recent studies have employed feature sets including time domain (AL-Salman et al., 2022), frequency domain (Hassan and Subasi, 2016), and chaos theory features (Nawaz et al., 2020) for EEG analysis. Zacharaki et al. (2013) proposes a two-step methodology based upon the fundamental morphological features of k-complex. In this method, the candidate waves are extracted, then it is confirmed as k-complex or not based on annotated k-complex. Krohne et al. (2014) develops a semi-automatic k-complex detection algorithm based on the wavelet transformation and various feature thresholds, achieving a positive true mean rate of 74% and a positive predictive value of 65%. Erdamar et al. (2012) presents an efficient algorithm combined wavelet and teager energy operator, it also relies on the amplitude and duration properties of the k-complex waveform. The results achieve up to 91% using ROC analysis. AL-Salman et al. proposes an ensemble model that combined fractal and frequency features based on dual-tree complex wavelet transform (DT-CWT) and achieves an average accuracy rate of 97.3% using three classification techniques (AL-Salman et al., 2019a). The researchers present an efficient method based on fractal dimension (FD) of time-frequency images coupled with undirected graph features. The results indicate that the proposed method yields better detection results with an average accuracy of 97% (AL-Salman et al., 2019b). Latreille et al. finds that awake-like EEG activity before the onset of k-complex followed by microarousals. They also indicate highlight region-specific sleep-or arousal-promoting responses following k-complex (Latreille et al., 2020). Tokhmpash et al. (2021) decomposes the EEG signals and then extracts various features from the sub-bands. The empirical results show the high efficiency of the proposed method in the analysis of EEG signals. Through a large number of k-complex detection approach based on features is proposed, systematic analysis of the relevance of the different features has not been fully carried out.

In addition to feature extraction, several classifiers have been used for the detection of k-complex. Vu et al. (2012) presents a k-complex detection method using hybrid-synergic machine learning, and the results based on tenfold cross-validation indicates that both the accuracy and the precision of this proposed model are at least as good as a human expert’s performance. The researchers develop an automatic k-complex detection method using a fuzzy neural network approach, which combines a fuzzy C-means algorithm and a neural network classifier (Ranjan et al., 2018). The paper detects the k-complex using a support vector machine based on amplitude and duration measurements, achieving a 91.40% of accuracy (Hernández-Pereira et al., 2016). Parekh et al. (2015) utilizes a fast non-linear optimization algorithm to detect k-complex, and achieves with F1 scores averaging 0.57 ± 0.02. Wessam et al. proposes a least square support vector machine (LS-SVM) classifier based on multi-domain features to identify k-complex obtaining average accuracy, sensitivity, and specificity of 97.7, 97, and 94.2%, respectively (AL-Salman et al., 2021). The detection algorithms typically can only detect EEG data into k-complex or non k-complex. However, it cannot deeply explore the complex relationships between features and k-complex or the underlying mechanisms of k-complex.

Apart from feature extraction and detection methods, feature selection also plays a crucial role in improving the performance of the considered task (Hernández-Pereira et al., 2016; Chen et al., 2022; Yang et al., 2022). As some of the extracted features may be redundant, feature selection is essential to remove the redundant and irrelevant features, which can decrease computational overhead (Hall, 1999; Dash and Liu, 2003; Zhao and Liu, 2007). In literature (Hancer et al., 2020), the research introduces a comprehensive survey on various feature selection methods from the perspective of clustering. Du et al. (2017) proposes a robust unsupervised method to remove redundant and irrelevant features. The results demonstrate that the proposed method performs better compared to some unsupervised feature selection methods. Yang and Nataliani (2018) presents the feature-reduction fuzzy c-means (FRFCM) by computing individual feature weight to reduce irrelevant feature components. The comparison of results demonstrate that FRFCM had good performance in terms of effectiveness and deficient ness in practice. Therefore, feature selection methods are employed to obtain a subset of features that accurately describe the given data and improve or maintain the detection performance and generalization capacity.

The primary intention of this study consists of: (1) to compare the influence of distinctive features and effective classifiers for k-complex detection; (2) to explore the effect of feature selection methods. Under those goals, we investigate and compare the ability of the detection k-complex based on time domain features, frequency domain features, and chaos theory features, etc.

The paper is organized as follows: the materials and methods are described in Section 2, which includes feature extraction, feature selection, and detection. In section 3, we present the experimental results and the relevant discussions. Conclusions are presented in Section 4.

## 2. Materials and methods

**Figure 1:**
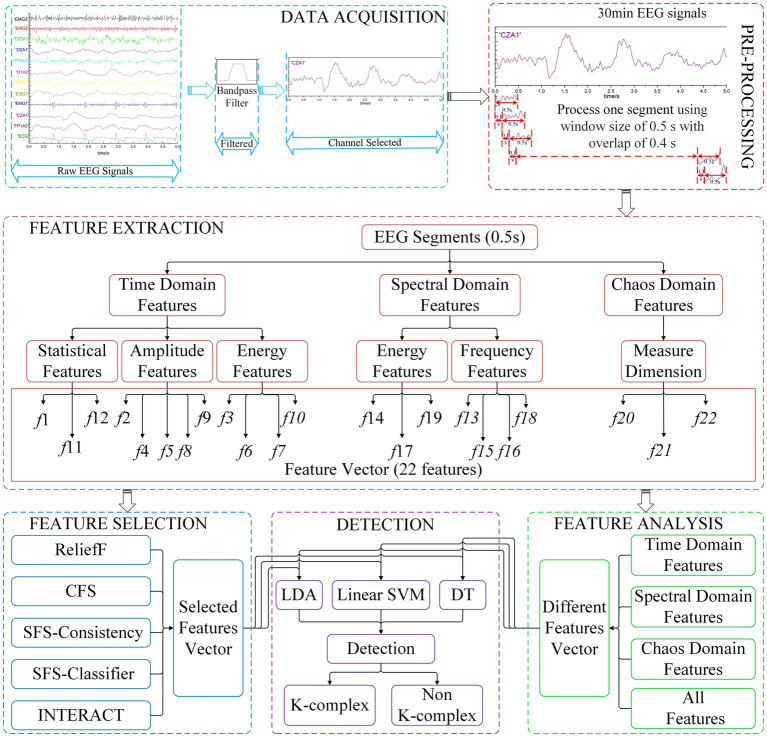
The k-complex detection flowchart of the proposed methods.

In the current research, the main objective is to develop an integrated method that yields optimal performance for k-complex detection task. Therefore, the pipeline of our experiments involves several key steps, namely data acquisition, pre-processing, feature extraction, feature selection, and detection methods. The original EEG signals are filtered and subsequently divided into segments using a sliding window technique. The window size is 0.5 s with overlap of 0.4 s. Then, the feature extraction methods are employed to calculate the feature vectors for each segment. The extracted features are served as the input to different feature selection methods. To evaluate the effectiveness, different classifiers are tested and compared. The detailed description is presented in subsection2.1 to subsection2.4. The entire flowchart of the proposed method is depicted in Figure 1.

### 2.1. Data acquisition and pre-processing

The EEG data used in this research is obtained from a sleep laboratory of a Belgium hospital.^1^ All sleep recordings acquired from ten subjects (28.1 ± 9.95 years old, which consist of 4 males and 6 females) is recorded with electrophysical signals such as electroencephalograph (EEG), electrooculograms (EOG), and electromyography (EMG) (Devuyst et al., 2010). The 30-min EEG signal is band-pass filtered with 4th order butterworth filter at 0.5 Hz to 30 Hz to smooth the raw signal and removed the environment noise caused by muscle activity and eye movement. In the current study, the Cz-A1 channel of EEG electrodes with the sampling frequency of 200 Hz is carried out to analyze.

**Figure 2:**
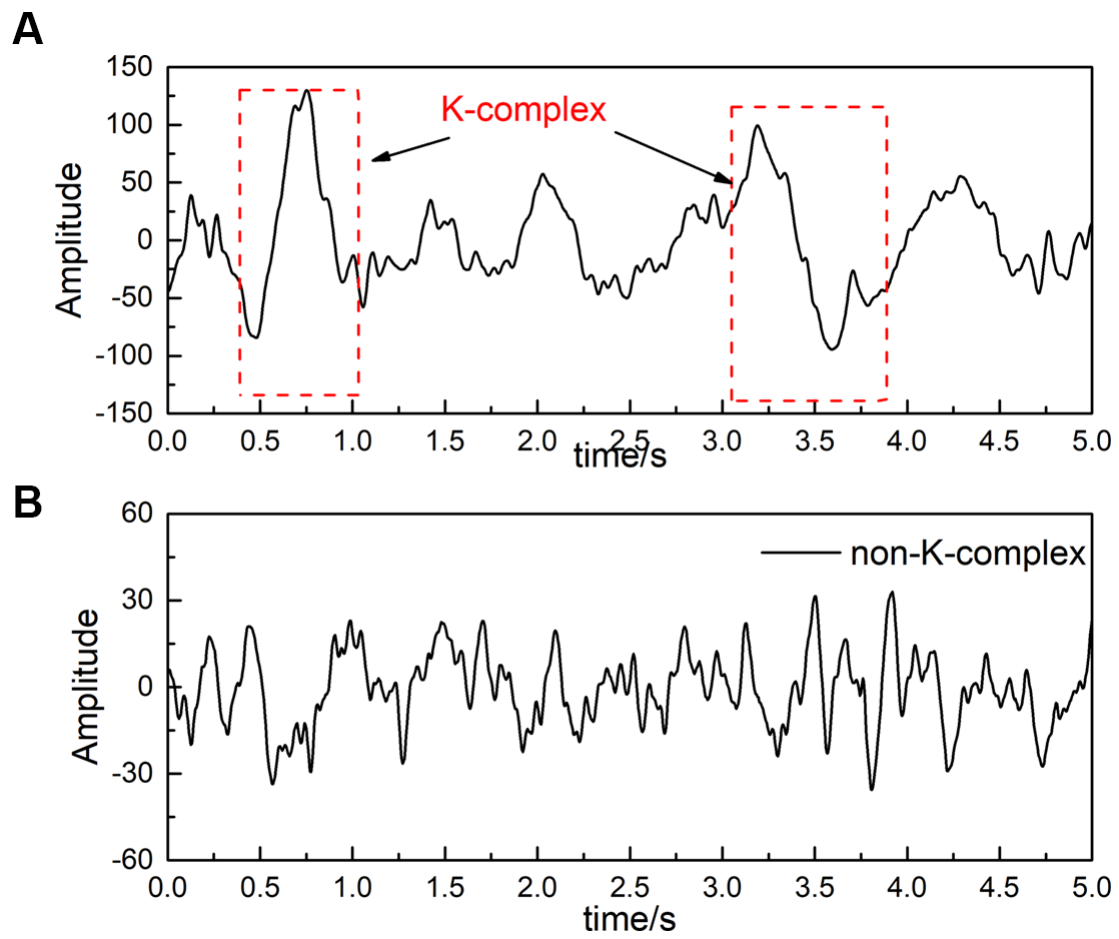
An filtered EEG signal [**(A)** is EEG signals with k-complex, and **(B)** represents EEG signals without k-complex].

For the DREAMS k-complex database, the recordings are given by two experts who independently score the k-complex according to the manual. In this paper, we choose the annotations marked by the expert 1 as a benchmark. Considering that k-complex wave last for about 0.5 s to 2 s, each 30-min EEG signals was divided into segments using the sliding window technique. The window size was selected as 0.5 s with a stepping of 0.1 s based on the previous studies (AL-Salman et al., 2019b, 2021). The feature vectors obtained from 0.5 s EEG segments are input classifiers to detect the k-complex and the non k-complex. The waves containing k-complex and non-k-complex were illustrated in Figure 2.

### 2.2. Feature extraction

The main objective during the feature extraction stages for k-complex detection system is to extract features from EEG segments that effectively capture the characteristics of the k-complex. Hence, finding a compact but effective set of features is deemed a crucial step in the k-complex detection task. The objective of this investigate is to evaluate the performance of different feature extraction schemes and find an optimal feature combination. Concretely, we analyze three different feature extraction methods, namely, time domain features, spectral domain features, and chaotic features. The first two feature sets consist of temporal and frequency features, which are listed in Tables 1, 2. The last one is based on chaotic features of EEG signals, which is listed in Table 2. All of these considered features explain in the following content.

**Table 1:** The features extraction of the k-complex defined by the time domain.

Numbers	Features	Formula
*f*1	Maximum value	$f_{\max}=\max\left(EEG\right)$
*f*2	Mean value	$f_{\mathrm{mean}}=\overset{N}{\underset{n=1}{\sum }}\mathrm{EEG}\left(n\right)/N$
*f*3	Standard deviation	$f_{std}=\sqrt{\frac{\sum_{n=1}^{N}{\left(EEG\left(n\right)-f_{mean}\right)}^{2}}{N-1}}$
*f*4	Skewness	$f_{skew}=E\left[{\left(\frac{EEG-f_{mean}}{f_{std}}\right)}^{3}\right]$
*f*5	Kurtosis	$f_{kurt}=E\left[{\left(\frac{EEG-f_{mean}}{f_{std}}\right)}^{4}\right]$
*f*6	Shape factor	$f_{shape}=\frac{\sqrt{\sum_{n=1}^{N}{\left(EEG\left(n\right)\right)}^{2}/N}}{\left(\sum_{n=1}^{N}\sqrt{\left|EEG\left(n\right)\right|}\right)/N}$
*f*7	Crest factor	$f_{crest}=\frac{\max\left(EEG\right)-\min\left(EEG\right)}{\sqrt{\sum_{n=1}^{N}{\left(EEG\left(n\right)\right)}^{2}/N}}$
*f*8	Impulse factor	$f_{impulse}=\frac{\max\left(EEG\right)-\min\left(EEG\right)}{\left(\sum_{n=1}^{N}\left|EEG\left(n\right)\right|\right)/N}$
*f*9	Margin factor	$f_{margin}=\frac{\max\left(EEG\right)-\min\left(EEG\right)}{{\left(\left(\sum_{n=1}^{N}\sqrt{\left|EEG\left(n\right)\right|}\right)/N\right)}^{2}}$
*f*10	Short energy	$f_{energy}=\overset{N}{\underset{i=1}{\sum }}{\left(EEG\left(n\right)\right)}^{2}$
*f*11	Zero-crossing Rate	$\begin{array}{c} \mathrm{sgn}\left[EEG\left(n\right)\right]=\{\begin{array}{cc} 1 & EEG\left(n\right)\geq 0\\ -1 & EEG\left(n\right)<0 \end{array}\\ num=\frac{1}{2}\overset{N}{\underset{n=2}{\sum }}\left|\mathrm{sgn}\left[EEG\left(n\right)\right]-\mathrm{sgn}\left[EEG\left(n-1\right)\right]\right|\\ f_{ZCR}=num/N \end{array}$
*f*12	Time centriod	$f_{TC}=\frac{\sum_{n=1}^{N}n.EEG\left(n\right)}{\sum_{n=1}^{N}EEG\left(n\right)}$

Where, EEG(n) , n=1,2,…,N , is the value of a time series. N is the number of data points in *EEG*.

**Table 2:** The spectral domain features and chaotic features of the k-complex.

	Numbers	Features	Formula	Explanation
Spectral features	*f* 13	Band Energy Ratio	$f_{BER}=\frac{\sum_{k=f_{1}}^{f_{2}}E\left(k\right)}{\sum_{k=1}^{K}E\left(k\right)}$	/
	*f* 14	Spectral Flux	$f_{SF}=\overset{K}{\underset{k=2}{\sum }}{\left|E\left(k\right)-E\left(k-1\right)\right|}^{2}$	/
	*f* 15	Spectral Centriod	$f_{SC}=\frac{\sum_{k=1}^{K}f_{k}.E\left(k\right)}{\sum_{k=1}^{K}E\left(k\right)}$	/
	*f* 16	Band Width of SC	$f_{BW}=\frac{\sum_{k=1}^{K}\left|f_{SC}-f_{k}\right|.E\left(k\right)}{\sum_{k=1}^{K}E\left(k\right)}$	/
	*f* 17	Spectral Flatness Measurement	$f_{SFM}=10.\log_{10}\left(\frac{{\left(\prod_{k=1}^{K}\left|E\left(k\right)\right|\right)}^{1/K}}{\left(1/K\right).\sum_{k=1}^{K}\left|E\left(k\right)\right|}\right)$	/
	*f* 18	Spectral Roll-off	$\begin{array}{c} \overset{f_{SRO}}{\underset{k=1}{\sum }}E\left(k\right)=C.\overset{K}{\underset{k=1}{\sum }}E\left(k\right)\\ C=0.6\sim 0.85 \end{array}$	/
	*f* 19	Spectral Irregularity	$f_{SI}=\frac{\sum_{k=2}^{K}{\left(E\left(k\right)-E\left(k-1\right)\right)}^{2}}{\sum_{k=1}^{K}{\left(E\left(k\right)\right)}^{2}}$	/
Chaotic features	*f* 20	Correlation Dimension	$CD=\underset{r\to 0}{\lim}\frac{\ln C\left(r\right)}{\ln r}$	C(r) represents the correlation integral
	*f* 21	Box Dimension	$BD=\underset{r\to 0}{\lim}\frac{\mathrm{lg}N\left(r\right)}{\mathrm{lg}\left(1/r\right)}$	N(r) represents the number of boxes
	*f* 22	Genealized Dimension	$GD=-\underset{r\to 0}{\lim}\frac{\mathrm{lg}\sum_{i}p_{i}^{q}}{\left(1-q\right).\mathrm{lg}r}$	$P=\left(p_{1},p_{2},\cdots p_{n},\right)$ means probability vector, and q is preselected parameter

Here, $f_{k}$ represents the frequency pin based on FFT, and E(k) represents the spectrum amplitude for EEG signals. $f_{1}$ is the first pin and $f_{2}$ is the last pin between selected band by scanning the frequency pin from left to right.

#### 2.2.1. Time features

Considering that the k-complex wave of EEG signals has a specifical changing trend in the time domain different from the non-k-complex wave, time domain features of EEG signals are determined (Günes et al., 2011; Lajnef et al., 2015). The widely-used time domain features in this paper are summarized in Table 1.

#### 2.2.2. Spectral features

Similar to time features, a variety of spectral analysis methods based on classical FFT are used to extract frequency domain features (Hassan and Bhuiyan, 2016; AL-Salman et al., 2018). Some traditionally ones are listed in Table 2.

#### 2.2.3. Chaotic feature

Considering that the characteristics of EEG signals are chaotic, not only the time domain or frequency domain features, the chaotic features are derived from nonlinear dynamical analysis, which are also applied to investigate the dynamic characteristics of EEG signals (Peker, 2016; AL-Salman et al., 2018). The details of chaotic features are described in Table 2.

### 2.3. Feature selection

Feature selection is another crucial issue in the detection system for k-complex, and its adoption is based on several reasons, which is as follows. Firstly, it can enhance the detection performance using the most relevant and informative features. Secondly, it is helpful in reducing costs and improve the efficiency of the detection process. Lastly, it facilitates effective discrimination and better understand of the relationship between k-complex and features. To estimate the quality of a feature selected, a correlation-based method was often utilized, the detection performance was also regarded as another evaluation criterion. In this paper, five feature selection methods, namely ReliefF, Correlation-based feature selection, Search-based feature selection (including two methods: consistency measures and classifier error rate measures), and INTERACT, are carefully explained and utilized to determine the effective feature subsets of the chosen dimensionality.

#### 2.3.1. ReliefF

**Figure 3:**
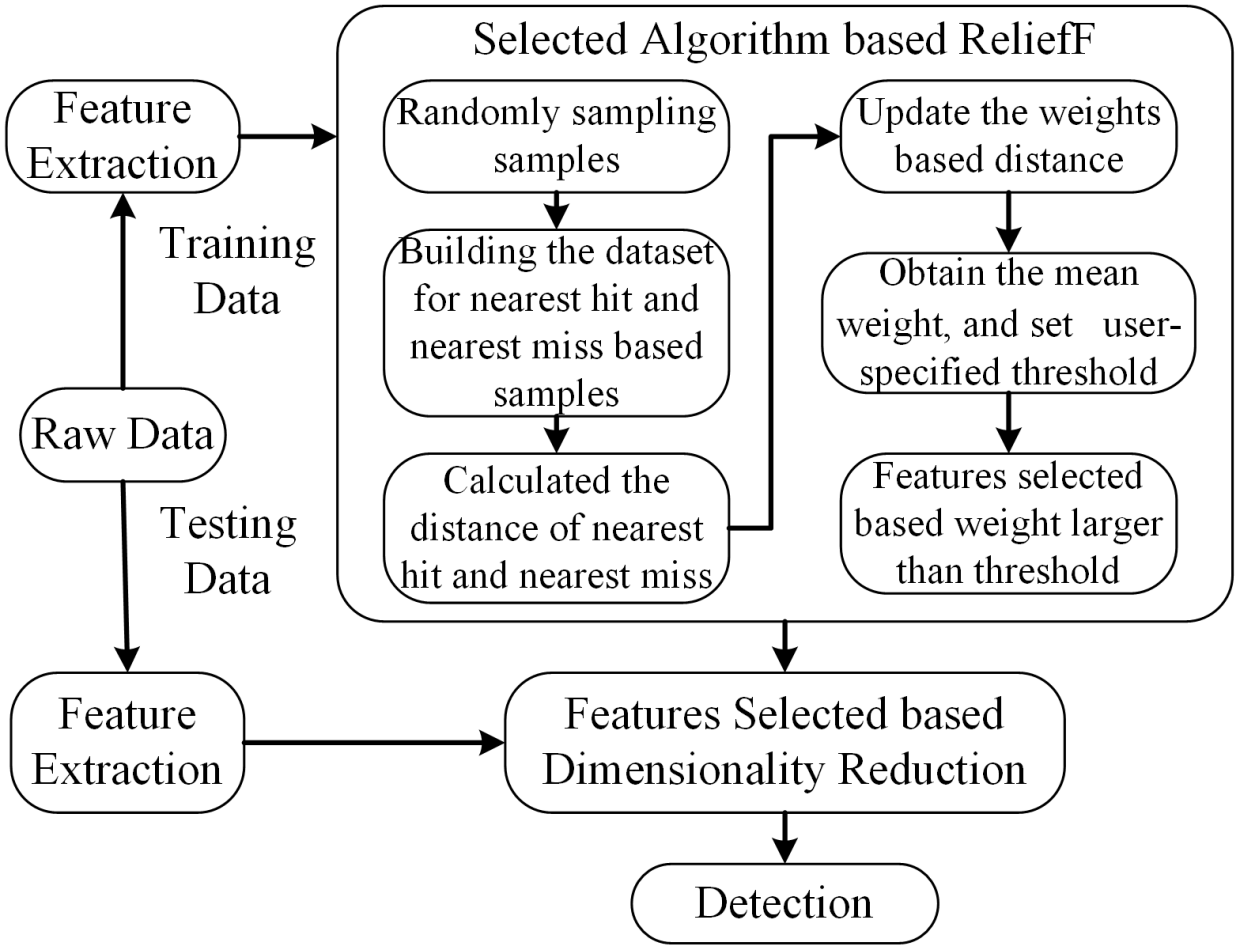
The scheme of ReliefF. The dimension of feature subsets is reduced based feature selected of ReliefF, and the selected features are used for further analysis.

ReliefF, as a feature weighting algorithm, calculates the weight describing the ability to draw a distinction of classes (Nawaz et al., 2020). Features are ranked based on weights, which are obtained according to the ability to distinguish the samples according to their distance in the feature space. If the weight of the feature is larger than a user-specified threshold (0.7 is selected in this paper), it will be selected to form the final feature subsets. The formula for updating the weight presented in Eq. 2.1.


(2.1) $$\begin{array}{l} Weight_{RF}=Weight_{RF}-\overset{k}{\underset{i=1}{\sum }}\frac{diff\left(f,SF,HF_{i}\right)}{num.k}+\\ \overset{k}{\underset{i=1}{\sum }}\frac{diff\left(f,SF,MF_{i}\right)}{num.k} \end{array}$$

where $Weight_{RF}$ represents the weight for features based ReliefF, num is sampling times, f denotes feature to calculate the weight, SF means near hits, which contained *k* cluster centroid belonging to the class of f , and MF means near misses, which contained k cluster centroid not belonging to the class of f . diff(f,A,B) is the distance between two samples ( A,B ) for a given feature ( f ). The scheme is illustrated in Figure 3.

#### 2.3.2. Correlation-based feature selection

Correlation-based feature selection (CFS) is a member of the most well-known and simplest filter algorithms (Hall, 1999). As a fully automatic algorithm, it does not determine or calculate any thresholds or the number of features selected. The weight of feature subsets is ranked according to the correlation based on the heuristic evaluation function. If the features are in a low correlation with the class, they will be regarded as irrelevant and ignored. And feature subsets are deemed as selected features if one is height association with the class among all feature combinations. The correlation can be derived from (Eq. 2.2).


(2.2) $$Metric_{CFS}=\frac{num.\bar{corr_{fl}}}{\sqrt{num+num.\left(num-1\right).\bar{corr_{ff}}}}$$

Here, $Metric_{CFS}$ is the metric coefficient based on CFS, num represents the number of feature subsets, $\bar{corr_{fl}}$ denotes the mean coefficient of correlation between the features and label variable, and $\bar{corr_{ff}}$ is the average correlation coefficient between feature subsets. The correlation coefficient in this method is Pearson’s correlation and all variables have been standardized.

**Figure 4:**
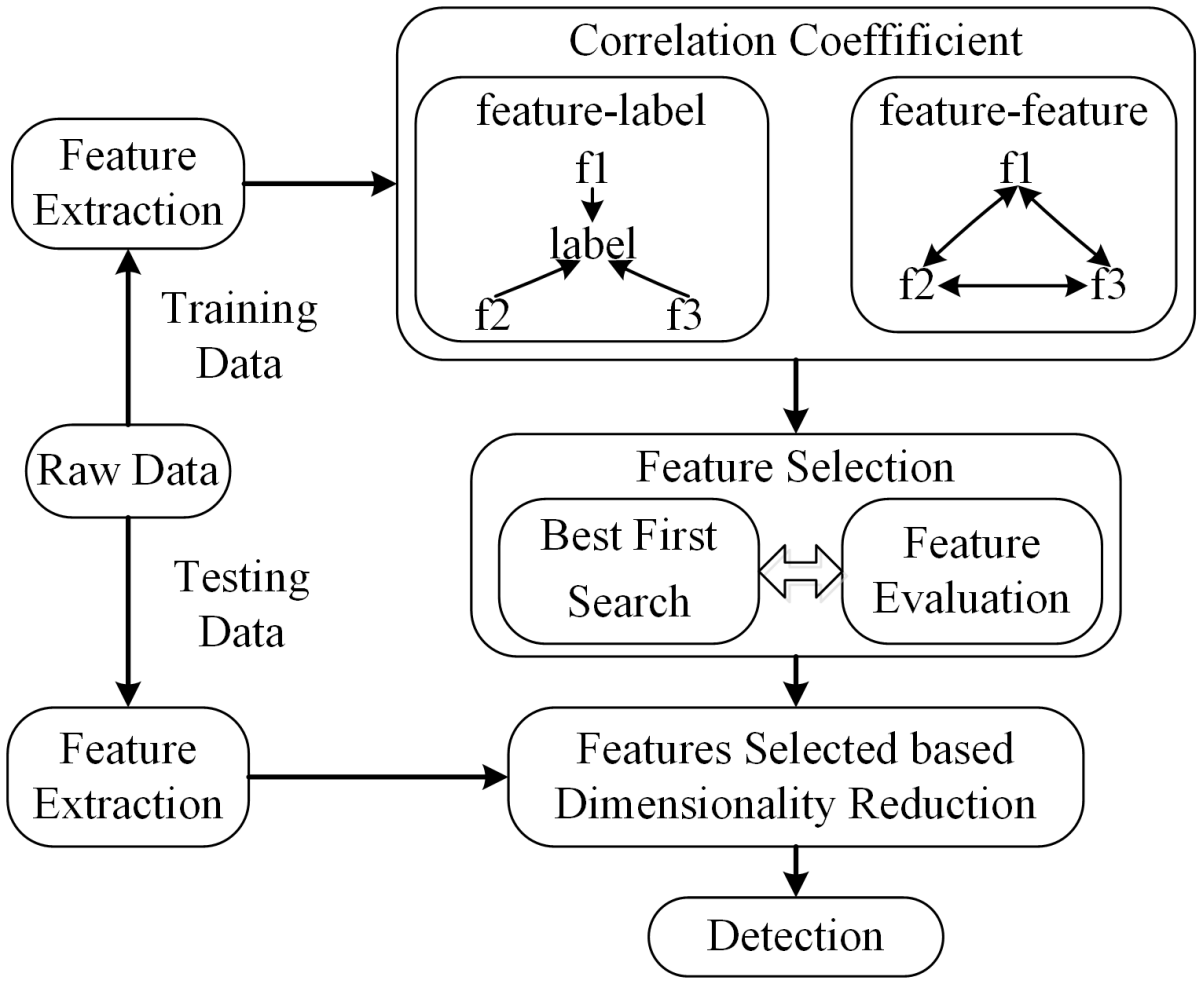
The scheme of CFS. The dimension of feature subsets is reduced based feature selected of CFS.

The features are extracted using raw training data. Then, the correlation coefficients between (feature, label) and (feature, feature) are calculated using Pearson’s correlation. The feature subsets are searched based on the best first search to find optimal features. The scheme of CFS is shown in Figure 4.

#### 2.3.3. Search-based feature selection

**Figure 5:**
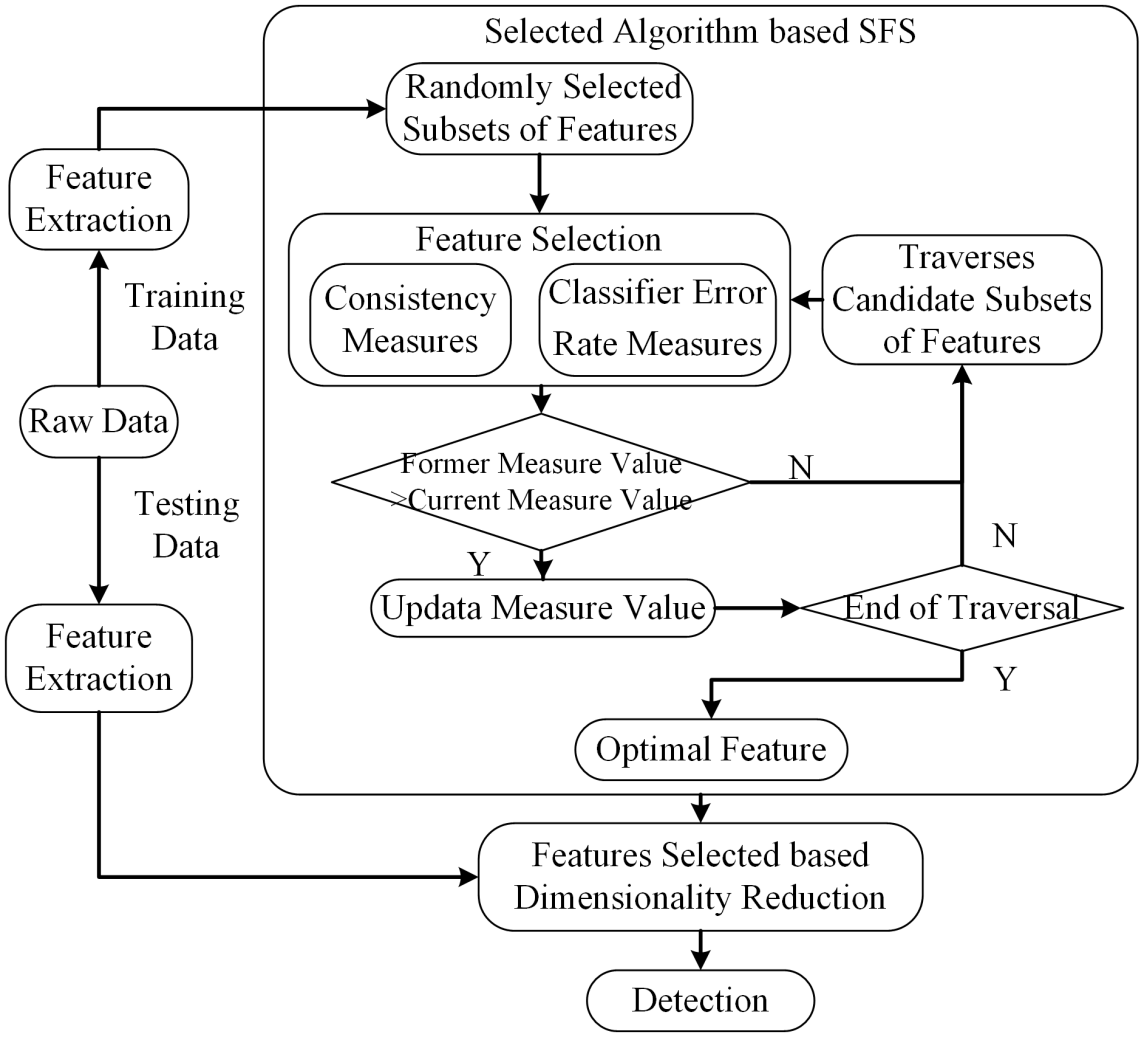
The scheme of SFS. The dimension of feature subsets is reduced based feature selected of SFS.

Search-based Feature Selection (SFS) method traverses all the subsets of features and tries to find the best performance among all candidate subsets based on some evaluation measures (Dash and Liu, 2003). Though this procedure needs to search all feasible subsets for features, it is not require the stopping criterion or pre-specified threshold. If the total number of features is N , $2^{N}$ denotes the number of all candidate subsets. The scheme of SFS is depicted in Figure 5. The algorithm is given as follows.

**Algorithm:** SFS.

**Input:** feature sets S , threshold δ.

**Output:** Feature subset T

1.
δ =0
2.
T=[]

/*traverses for all features subsets*/

1. for all features combination $S_{i}$ in S
2.
$\delta_{i}$ =thresholdCal( $S_{i}$ )
3. if $\delta_{i}<\delta$
4.
$\delta =\delta_{i}$ and $T=S_{i}$

Return T

We employ two kinds of evaluation measures to evaluate the ability based on subset features to distinguish the different classes. The details of each evaluation measures are presented in the following paragraph.

##### 2.3.3.1. Consistency measures

The consistency-based filter evaluates the attributes of selected features according to the inconsistency rate (Zhao and Liu, 2007). If the inconsistency rate of current selected features falls below the pre-selection features, current selected features are regarded as the selected features. The consistency measures was achieved as follows:

Step 1: The discrimination of inconsistent instances. If $d_{i}$ and $d_{j}$ are feature vectors of two instances with identical values except for their class labels, they are considered as one inconsistent instances.

Step 2: Inconsistency count. The set of inconsistent-instances is divided into 2 groups (k-complex or non-k-complex) based on their corresponding class label. The inconsistency count is calculated by subtracting the largest count of different class labels from the number of occurrences of a feature in the data.

Step 3: Inconsistency rate. The inconsistency rate is obtained by dividing the sum of all the inconsistency counts in a feature subset by the total number of patterns.

##### 2.3.3.2. Classifier error rate measures

As one of the traditional wrapper methods, the classifier error rate measures selected feature subsets according to the predicting accuracy. The selected feature is updated if the accuracy level is higher.

#### 2.3.4. Interact

Let us consider the situation that some features might have a wake correlation with the labels, while the features may be a strong correlation if they were combined with other features. For this case, the INTERACT method is proposed to search for interacting features (Zhao and Liu, 2007). As a filter-based method, it employs the backward search strategy to remove the features deemed as irrelevant.

**Figure 6:**
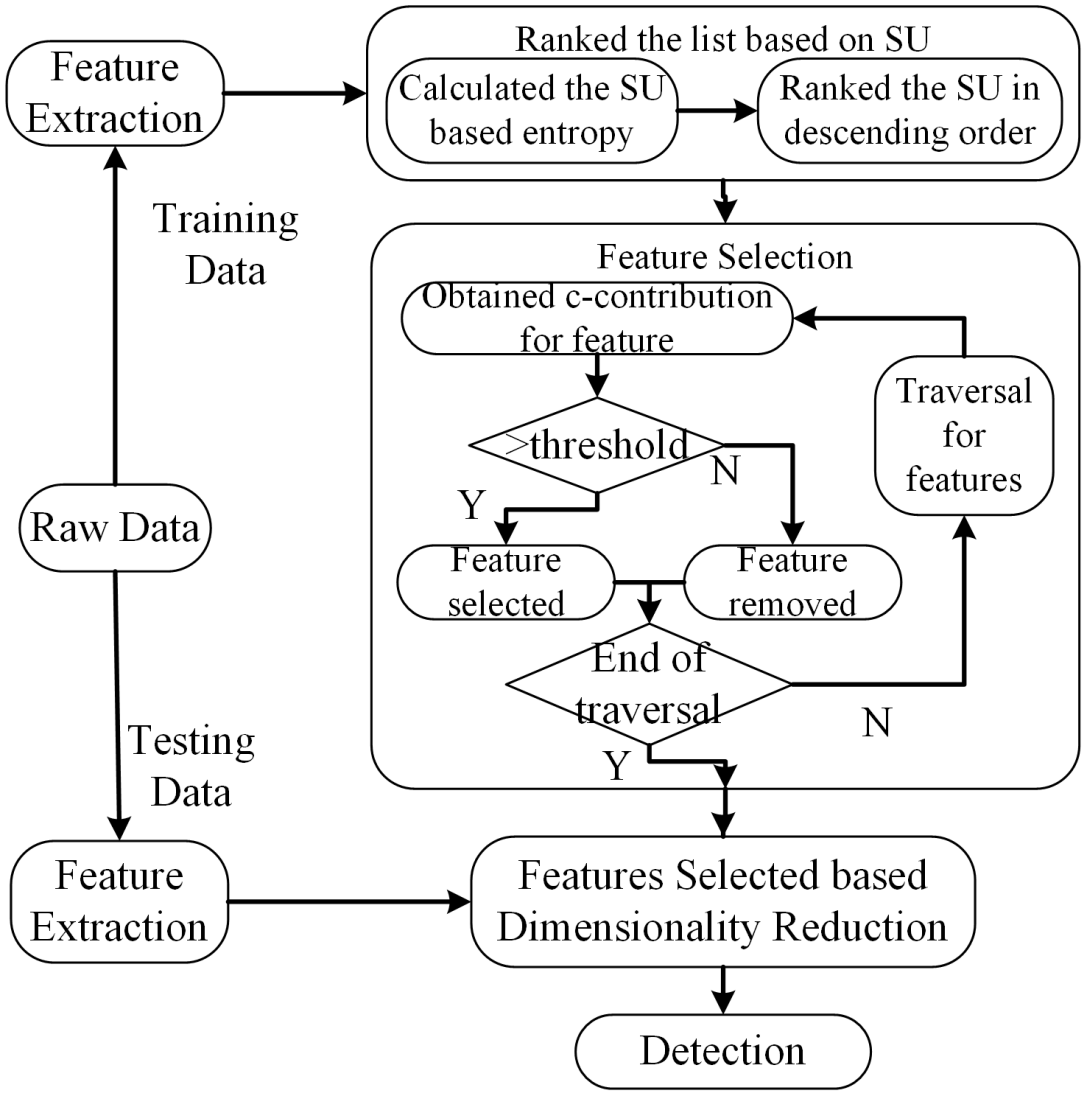
The scheme of INTERACT method. The dimension of feature subsets is reduced based feature selected of INTERACT.

The core parts employed by this method can be divided into two steps. The details are illustrated in Figure 6. In the first step, the features are ranked in the descending order taken into account their symmetrical uncertainty (SU) values. Let l denotes the class label and f means the feature vector. Let H(f) and H(f,l) denote entropy and joint entropy, respectively. The SU between features and labels can be derived from Eq. 2.4. In the second step, features are evaluated using consistency contribution (c-contribution) from the end of the ranked feature listed in the first step. Once the c-contribution is larger than the specific threshold, the feature will be selected, otherwise, it is removed.


(2.4) $$\mathrm{SU}=2\times \left[\frac{H\left(f\right)+H\left(l\right)-H\left(f,l\right)}{H\left(f\right)+H\left(l\right)}\right]$$

### 2.4. Detection

Feature combinations obtained from the feature selection techniques are further assessed using classifiers under 5-fold cross-validation. In this paper, the detection algorithms in Figure 1 are listed: Linear Discriminant Analysis (LDA) tries to maximize the ratio of the between-class variance and the within-class variance. Typically, LDA is generally used to classify patterns between two classes; linear support vector machine (SVM) is a statistical classification algorithm, which has been widely used in many recognition fields; and decision tree (DT), which has a fast computation time for a real-time system and a strong interpretation ability for features, has also a promising result.

### 2.5. Performances metrics

In all used methods, the adopted metrics including sensitivity, specificity, and average classification accuracy can be calculated from the confusion matrix determined by the parameters presented in Table 3.

**Table 3:** Confusion matrix.

		Predicted k-complex	
		yes	no
Actual k-complex	Yes	True positive (TP)	False negative (FN)
	No	False positive (FP)	True negative (TN)

The sensitivity, specificity, and average classification accuracy are calculated from the equation as follows.

Sensitivity is also called as true positive rate, measuring the proportion of the actual positive predication and estimates the performance of the detection method.


(2.4) $$\mathrm{sensitivity}=\frac{TP}{TP+FN}\;$$

Specificity is called true negative rate, measuring the proportion of the actual negative prediction and also reflects the performance of the classification method.


(2.5) $$\mathrm{specificity}=\frac{TN}{TN+FP}$$

Accuracy indicates the rate of rightly classified cases.


(2.6) $$\mathrm{accuracy}=\frac{TP+TN}{TP+FN+FP+TN}$$

$F_{score}$ , as one of the most important measurements for detection, is used to reflect the importance between the rate of true k-complex and the detected k-complex. β>1 represents that we focus on the rate of true k-complex, and if 0<β<1 , the rate of detecting k-complex has a larger influence.


(2.7) $$F_{score}=\frac{\left(1+\beta^{2}\right)\times TP}{\left(1+\beta^{2}\right)\times TP+\beta^{2}\times FN+FP}$$

Kappa coefficient is generally used to evaluate the agreement between two classification results. In this paper, it is employed to evaluate the agreement between the different feature selection model and the detection model. It can be defined as Eq. 2.8.


(2.8) $$kappa=\frac{accuracy-\frac{\begin{array}{l} \left(TP+FN\right)\left(TP+FP\right)+\\ \left(FP+TN\right)\left(FN+TN\right) \end{array}}{{\left(TP+FN+FP+TN\right)}^{2}}}{1-\frac{\begin{array}{l} \left(TP+FN\right)\left(TP+FP\right)+\\ \left(FP+TN\right)\left(FN+TN\right) \end{array}}{{\left(TP+FN+FP+TN\right)}^{2}}}$$

Here, *TP* represents the number of k-complex marked by experts and also predicts k-complex. *TN* is the number of non-k-complex labeled by experts and can be detected as non-k-complex using our proposed method. *FN* means that the number of k-complex is marked by experts but detects as non-k-complex. *FP* is the number of non-k-complex labeled by experts but is predicted as k-complex.

J1 Value analyzes the separability according to the Fisher criteria, which can illustrate the effectiveness of features. The calculation of J1 value is obtained from Eq. 2.9.


(2.9) $$J_{1}=\mathrm{tr}\left(S_{w}^{-1}S_{m}\right)$$

Here, $S_{w}$ and $S_{m}$ represent the within-class and between-class scatter matrix, respectively. tr(S) means the trace of square matrix S.

## 3. Results and discussion

Considering that 22 features are defined to represent the k-complex detection, we will perform a comparison on these feature vectors. For the convenience of comparison, the experiments are conducted to evaluate the performance of features. We calculate the spearman correlation coefficient, significance, and J1 values between different feature sets. Besides, detection ability of different features is also reported to capture the distinctness between k-complex and non k-complex.

### 3.1. evaluating the effectiveness of the feature sets

**Figure 7:**
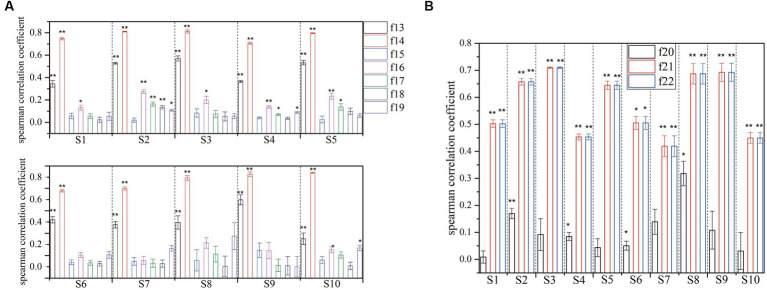
Correlation coefficient and significance test for the different domain features with features of k-complex and non-k-complex (if the value of p below than 0.005, it is marked with **, and if the *p*-value is below than 0.05, it is marked with *. **(A)** is for spectral feature, and **(B)** is for chaotic features).

To illustrate the effectiveness of different types of features, we adopt the spearman correlation coefficient and significance as the metrics in this experiment. The results are shown in Table 4; Figure 7. A larger correlation coefficient suggests a better feature for the detecting of k-complex. Additionally, value of p is also calculated using a one-way analysis of variance to verify statistically significant between k-complex and non-k-complex. The statistical results show that the performance of various types of features to detect k-complex are significantly difference with *p* < 0.05.

**Table 4:** Correlation coefficient and significance test for the time domain features with features of k-complex and non-k-complex.

No. of features	Correlation coefficient	*p*-value	Result
**f1**	**0.7488**	**0.0000**	**S**
**f2**	**0.7983**	**0.0000**	**S**
f3	0.1921	0.1212	NS
f4	0.1447	0.2345	NS
f5	0.1801	0.1136	NS
f6	0.1427	0.2611	NS
f7	0.4682	**0.0002**	NS
f8	0.4303	**0.0025**	NS
f9	0.3914	**0.0087**	NS
**f10**	**0.8122**	**0.0000**	**S**
f11	0.5147	**0.0006**	NS
f12	0.1852	0.1097	NS

If value of *p* < 0.05 and correlation coefficient > 0.6 significant(S); otherwise not significant (NS). It is also noted that the features with statistically significant are highlighted in bold.

Independent sample test of time domain features between k-complex and non-k-complex is presented in Table 4. As seen from the Table 4, the features {*f*1, *f*2, *f*10}, highlighted in bold, have a high correlation coefficient (>0.7) and the difference is statistically significant (*p* < 0.05). Meanwhile, there are significant differences in detecting the k-complex for features {*f*7, *f*8, *f*9, *f*11} with a moderate level of relation. On the contrary, the statistical test shows no significant differences for other features.

Individually, the significance analysis and the correlation coefficient with different subjects are also investigated, which is displayed in Figure 7. Seven spectral features and three chaotic features are extracted from each segment, and then are fed to verify the statistical test. Obviously, the feature {*f*14} exhibits a high correlation with mean value of 0.7709, while the feature {*f*13} shows a moderate level of relation with a mean value of 0.4379. However, it is clear that the remaining spectral features have low correlation. In terms of significance analysis, a further test reveals a significant difference for features {*f*13, *f*14} with *p* < 0.005. Additionally, features {*f*16, *f*17} have low correlation, while some of subjects exhibited significant difference with *p* < 0.05. On the other hand, no significant differences are found for others. Furthermore, the results of chaotic features analysis are presented in Figure 7B. A hypothesis test using value of p is conducted to determine the significant features in this regard. The significance level of features {*f*21, *f*22} is found to be smaller than 0.005, indicating a significant difference between different features sets. Moreover, these features demonstrate a high or moderate level of relation. Based on the results, the extracted features {*f*13, *f*14, *f*21, *f*22}, using the statistical test method, achieve higher values compared with other features.

**Figure 8:**
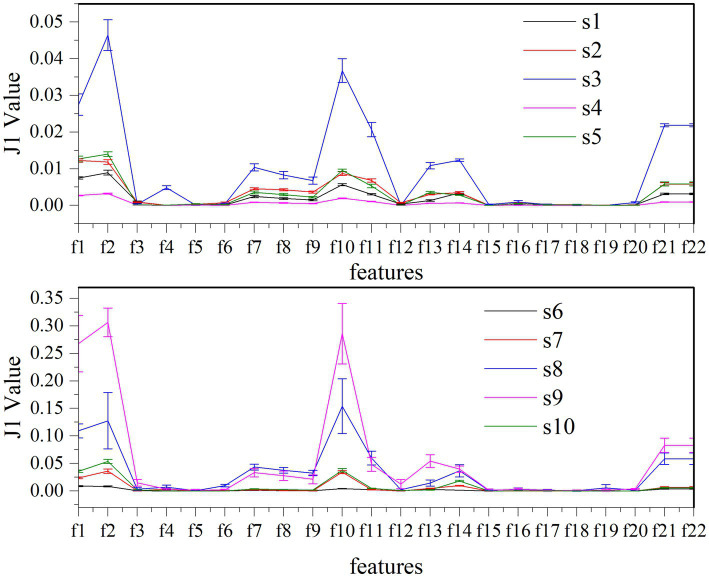
Comparison of J1 value between different features and subjects for k-complex detection.

Twenty-two features are tested to evaluate the effectiveness of k-complex detection in EEG signals. The separability of these different features are assessed based on J1 value in this experiment. The higher J1 value indicates greater separability between features. The value of J1 value and comparison for different features is presented in Figure 8. According to the obtained results, it is evident that the features {*f*1, *f*2, *f*10, *f*11, *f*13, *f*14, *f*21, *f*22} have achieved higher J1 values, which indicates that these features effectively characterize the k-complex. This finding is consistent with the inferences drawn from Table 4; Figure 7. Furthermore, it is worth noting that significant differences exist between the results obtained from the time domain features and the chaotic features.

### 3.2. Methodology comparison based different types of features

**Figure 9:**
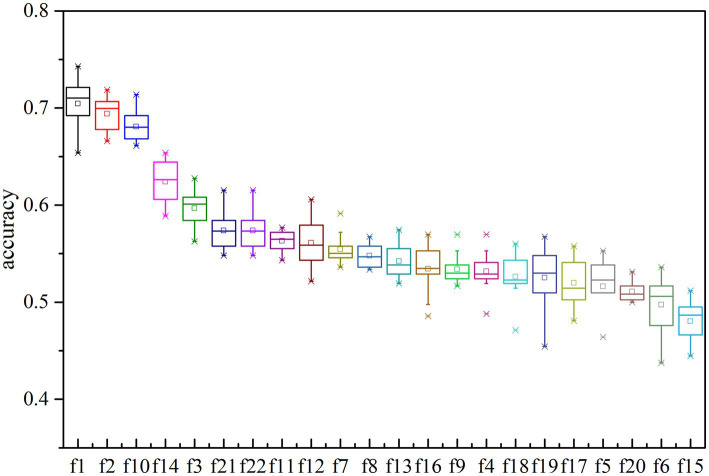
The accuracy of multi-domain features based on decision trees model for each features. Each box represents the 25–75th percentiles, and central line is the median value, the tiny vertical lines extend to the most extreme data not considering as outliers, which are plotted individually.

To evaluate the effectiveness of each feature type for k-complex detection, a single feature is utilized at a time to determine which features effectively reflect the presence k-complex. The detection accuracy is then tested and conducted by performing the traditional machine learning algorithm. The results are presented in Figure 9. All the features are ranked and sorted in descending order based on their importance in terms of detection accuracy. It is evident that the top-ranked features vectors {*f*1, *f*2, *f*3, *f*7, *f*10, *f*11, *f*12, *f*14, *f*16, *f*21, *f*22} perform better than the others to reflect the distinctness of k-complex. This result is consistent with the previous finding shown in Table 4, especially for the highly correlated feature vectors {*f*1, *f*2, *f*10}. These evidence indicate that the time features are slightly more important than spectral features and chaotic features. Furthermore, it is also observed that the highest accuracy for k-complex detection reaches a maximum value, with an average accuracy of 74.28%. This also verifies the conclusion that the proposed feature vectors have the potential to capture the characteristics of k-complex, and can contribute to the k-complex detection task, effectively.

**Figure 10:**
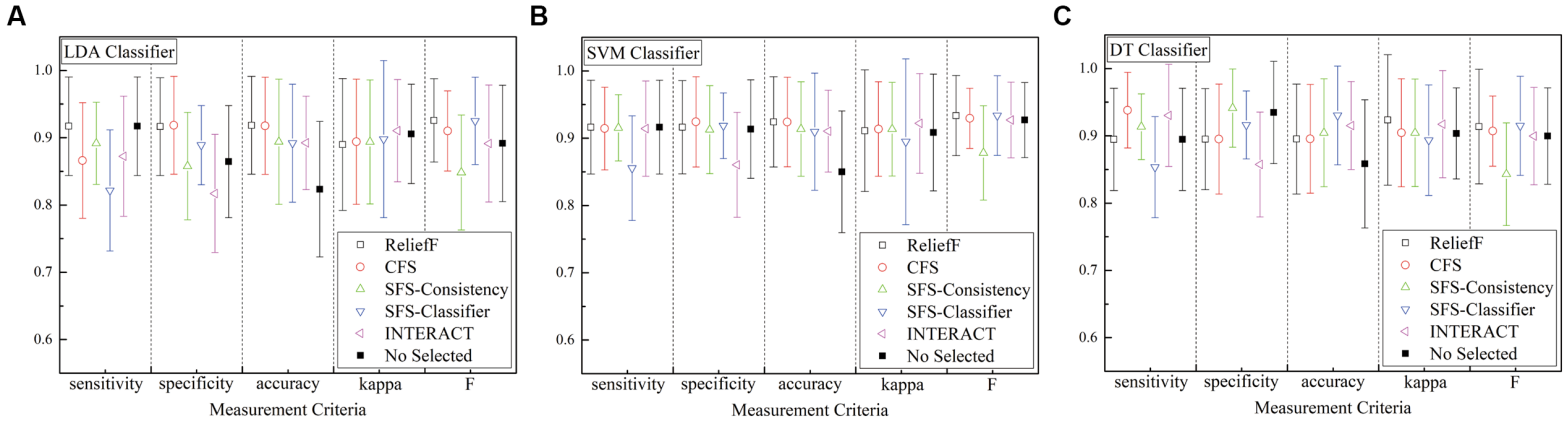
Comparison of feature selection methods and without feature selection method using three detection algorithm **(A)** is for LDA algorithm, **(B)** is for LSVM algorithm, and **(C)** is for DT algorithm. No selected means that without any feature selection process.

Based on the previous description, the results of different features are obtained from several detection methods for the k-complex detection. For the sake of clarity, the results are analyzed in terms of accuracy, sensitivity, specificity, kappa coefficient, and F-scores, which are presented in Figure 10. From the figure, all of the accuracy results obtain by the feature selection models outperform than that achieved without any feature selection process for any of the detection algorithm, which is 82.36 ± 10.06%, 85.01 ± 9.04%, and 85.85% ± 9.52% for LDA, LSVM, and DT detection algorithm, respectively. In particular, the feature selection method of the SFS-classifier achieved the best accuracy with 93.03% ± 7.34% using the DT algorithm. In the case of LSVM and DT detection algorithm, the sensitivity of SFS-classifier achieved is slightly worse than without the feature selection process, and other methods obtained better results compared with the methods without feature selection process. The model combined CFS and DT is the best option for sensitivity of 93.81 ± 5.62%. From the view of kappa, the performance of feature selection methods have similar results for LDA, and for other classifiers, the results are slightly higher than methods without the feature selection process. Among the feature selection models being tested, the application of feature selection methods turns out to have better performance than that without any feature selection process. These results confirm that the performance of the k-complex detection task is better when using feature selection, although there is no classifier that clearly outperforms the others.

### 3.3. Comparison with the existing methods based the same database

To evaluate the performance of the proposed methods, we compare the proposed method with other existing methods of the k-complex detection. All the selected studies are conducted using the same databases as described in subsection 2.1. Table 5 presents the comparisons among the proposed method and others. A semi-automatic k-complex detection algorithm-based wavelet transformation is proposed to identify pseudo k-complex and reject false positives using the feature threshold method, and achieves a mean sensitivity of 74% (Krohne et al., 2014). A fuzzy decision combined with hjorth parameters is proposed, and the average sensitivity and specificity of 86 and 82% are achieved compared to the visual human scoring (Migotina et al., 2010). Ranjan et al. (2018) proposes a fuzzy neural network approach to detect the k-complex, the results show that an average of accuracy and specificity of 87.65 and 76.2%, respectively. The algorithm to extract fractal dimension based on the box counting method is used to detect the k-complex, achieving the average accuracy, sensitivity, and specificity of 91, 87, and 92% (AL-Salman et al., 2019b). A Multitaper-based k-complex detection method is proposed by Oliveira et al. (2020), and a mean sensitivity of 85.1% is achieved. An efficient method for k-complex detection algorithm coupled with a RUSBoosted tree model is presented, and achieving the average accuracy, sensitivity, and specificity of 92.18, 92.41, and 92.41%, respectively (Li and Dong, 2023). In general, the proposed method is more excellent than others in almost all metrics. According to those comparison, it is clearly demonstrated that the proposed method obtains an acceptable performance and is effective and suitable to detect k-complex in EEG signals.

**Table 5:** Performance comparisons between the proposed method and other different detection methods with the same datasets.

Methods	Accuracy (%)	Sensitivity (%)	Specificity (%)
Semi-automatic k-complex detection algorithm based wavelet transformation	/	74	/
A fuzzy decision combined with hjorth parameters	/	86%	82%
Fuzzy neural network approach	87.65	**/**	76.2
Box counting method	91%	87%	92%
Multitaper-based method	/	85.1	/
RUSBoosted trees method based TQWT	92.18	92.41	92.41
Proposed methods	**93.03±7.34**	**93.81±5.62**	**94.13±5.81**

It is noted that the best performance are highlighted in bold compared with other methods.

## 4. Conclusion

This paper focuses on evaluating the performance of multi-domain features, feature selection methods, and detection algorithms in the detection of k-complex based on EEG signals. The suitable combinations of these techniques have the potential to improve the development of sleep analysis. Most existing papers compare single feature extraction methods, single selection algorithm, or a single detection algorithm. Few works comprehensively compare for the entire process including feature extraction, selection and detection. Hence, this paper aims to provide a comprehensive analysis of k-complex detection from this perspective.

Considering that k-complex is a high-amplitude wave compared to the relatively low background activity of the N2 sleep stage, and the samples were taken only from the N2 stage in some situations. However, it should be noted that detecting k-complex from N3 stages (slow waves with high amplitude, which is similar to k-complex) may have some disadvantages. At the same time, even though the number of N3 makes up a small proportion compared with other sleep stages, it also has some influence on the overall performance. Therefore, the sliding window technique is utilized to segment the whole EEG signals. The results demonstrate that when feature selection methods are applied, there is a significant improvement in performance compared to the performance without feature selection process. Additionally, feature selection methods can effectively decrease the dimensionality of the features.

It is believed that the methods described in this paper may turn out to be useful for investigating the k-complex. Moreover, the utility of feature selection methods and detection models illustrates that k-complex detection and feature analysis are more interesting. Furthermore, these results also denoted that combinations of techniques can be employed in real-time detection of EEG signals. It is worthwhile that further discuss on the principles of feature selection methods in our future studies.

## Data availability statement

The datasets presented in this study can be found in online repositories. The names of the repository/repositories and accession number(s) can be found at: https://zenodo.org/record/2650142.

## Author contributions

YL conceived and designed the experiments. YL and XD performed the experiments and analyzed the data. YL and KS wrote and revised the paper. XB and FK revised the paper. YL and HL provided the funding support. All authors contributed to the article and approved the submitted version.

## Funding

Funding was provided from Natural Science Basic Research Program of Shaanxi Program (2022JQ-598), scientific research plan projects of Shaanxi Education Department (20JK0917), National Natural Science Foundation of China (62202378), and Doctoral Scientific Research Starting Foundation of Xi’an University of Posts and Telecommunications (315020018). This paper also obtained the support from Shaanxi key disciplines of special funds to finance projects.

## Conflict of interest

The authors declare that the research was conducted in the absence of any commercial or financial relationships that could be construed as a potential conflict of interest.

## Publisher’s note

All claims expressed in this article are solely those of the authors and do not necessarily represent those of their affiliated organizations, or those of the publisher, the editors and the reviewers. Any product that may be evaluated in this article, or claim that may be made by its manufacturer, is not guaranteed or endorsed by the publisher.

## Footnotes

^1^ https://zenodo.org/record/2650142

## References

[ref1] AL-Salman W. Li Y. Oudah A. Y. Almaged S. (2022). Sleep stage classification in EEG signals using the clustering approach based probability distribution features coupled with classification algorithms. Neurosci. Res. 188, 51–67. doi: 10.1016/j.neures.2022.09.009, PMID: 36152918

[ref2] AL-Salman W. Li Y. Wen P. (2019a). K-complexes detection in EEG signals using fractal and frequency features coupled with an ensemble classification model. Neuroscience 422, 119–133. doi: 10.1016/j.neuroscience.2019.10.034, PMID: 31682947

[ref3] AL-Salman W. Li Y. Wen P. (2019b). Detection of EEG k-complexes using fractal dimension of time frequency images technique coupled with undirected graph features. Front. Neuroinform. 13, 1–19. doi: 10.3389/fninf.2019.00045, PMID: 31316365 PMC6609999

[ref4] AL-Salman W. Li Y. Wen P. (2021). Detection of k-complexes in EEG signals using a multi-domain feature extraction coupled with a least square support vector machine classifier. Neurosci. Res. 172, 26–40. doi: 10.1016/j.neures.2021.03.012, PMID: 33965451

[ref5] AL-Salman W. Li Y. Wen P. Diykh M. (2018). An efficient approach for EEG sleep spindles detection based on fractal dimension coupled with time frequency image. Biomed. Signal Process. Control 41, 210–221. doi: 10.1016/j.bspc.2017.11.019

[ref6] Berry R. B. Brooks R. Gamaldo C. E. Harding S. M. Lliyd R. M. Marcus C. L.. (2015). "The AASM manual for the scoring of sleep and associated events, rules, terminology and technical specification," American Academy of Sleep Medicine. Darien, IL.

[ref7] Chen K. Hangjun C. Li X. Leung M. F. (2022). Graph non-negative matrix factorization with alternative smoothed L0 regularizations. Neural Comput. & Applic. 35, 9995–10009. doi: 10.1007/s00521-022-07200-w

[ref8] Dash M. Liu H. (2003). Consistency-based search in feature selection. Artif. Intell. 151, 155–176. doi: 10.1016/S0004-3702(03)00079-1

[ref9] Devuyst S. Dutoit T. Stenuit P. Kerkhofs M. (2010). Automatic k-complexes detection in sleep EEG recordings using likelihood thresholds. Conference of the IEEE engineering in medicine and biology society. Buenos Aires. Argentina. 10.1109/IEMBS.2010.5626447 21096240

[ref10] Du S. Ma Y. Li S. Ma Y. (2017). Robust unsupervised feature selection via matrix factorization. Neurocomputing 241, 115–127. doi: 10.1016/j.neucom.2017.02.034

[ref11] Dumitrescu C. Costea I.-M. Cormos A.-C. Semenescu A. (2021). Automatic detection of k-complexes using the cohen class recursiveness and reallocation method and deep neural networks with EEG signals. Sensors 21, 1–19. doi: 10.3390/s21217230, PMID: 34770537 PMC8587652

[ref12] Erdamar A. Duman F. Yetkin S. (2012). A wavelet and teager energy operator based method for automatic detection of K-complex in sleep EEG. Expert Syst. Appl. 39, 1284–1290. doi: 10.1016/j.eswa.2011.07.138

[ref13] Günes S. Dursun M. Polat K. Yosunkaya S. (2011). Sleep spindles recognition system based on time and frequency domain features. Expert Syst. Appl. 38, 2455–2461. doi: 10.1016/j.eswa.2010.08.034

[ref14] Hall M. A. (1999). Correlation-based feature selection for machine learning, The University of Waikato. Hamilton

[ref15] Hancer E. Xue B. Zhang M. (2020). A survey on feature selection approaches for clustering. Artif. Intell. Rev. 53, 4519–4545. doi: 10.1007/s10462-019-09800-w

[ref16] Hassan A. R. Bhuiyan M. I. H. (2016). A decision support system for automatic sleep staging from EEG signals using tunable Q-factor wavelet transform and spectral features. J. Neurosci. Methods 271, 107–118. doi: 10.1016/j.jneumeth.2016.07.012, PMID: 27456762

[ref17] Hassan A. R. Bhuiyan M. I. H. (2017). An automated method for sleep staging from EEG signals using normal inverse gaussian parameters and adaptive boosting. Neurocomputing 219, 76–87. doi: 10.1016/j.neucom.2016.09.011

[ref18] Hassan A. R. Subasi A. (2016). Automatic identification of epileptic seizures from EEG signals using linear programming boosting. Comput. Methods Prog. Biomed. 136, 65–77. doi: 10.1016/j.cmpb.2016.08.013, PMID: 27686704

[ref19] Hernández-Pereira E. Bolón-Canedo V. Sánchez-Maroño N. Álvarez-Estévez D. Moret-Bonillo V. Alonso-Betanzos A. (2016). A comparison of performance of K-complex classification methods using feature selection. Inf. Sci. 328, 1–14. doi: 10.1016/j.ins.2015.08.022

[ref20] Horie K. Ota L. Miyamoto R. Abe T. Suzuki Y. Kawana F.. (2022). Automated sleep stage scoring employing a reasoning mechanism and evaluation of its explainability. Sci. Rep. 12:12799. doi: 10.1038/s41598-022-16334-9, PMID: 35896616 PMC9329306

[ref21] Khasawneh N. Fraiwan M. Fraiwan L. (2022). Detection of K-complexes in Eeg waveform images using faster R-Cnn and deep transfer learning. BMC Med. Inform. Decis. Mak. 22, 1–14. doi: 10.1186/s12911-022-02042-x, PMID: 36397034 PMC9673349

[ref22] Krohne L. K. Hansen R. B. Christensen J. A. E. Sorensen H. B. D. Jennum P. (2014). "detection of K-complexes based on the wavelet transform", in: 36th annual international conference of the IEEE engineering in medicine and biology society. (Chicago, IL, USA: IEEE). 10.1109/EMBC.2014.6944859 25571227

[ref23] Lajnef T. Chaibi S. Eichenlaub J.-B. Ruby P. M. Aguera P.-E. Samet M.. (2015). Sleep spindle and k-complex detection using tunable Q-factor wavelet transform and morphological component analysis. Front. Hum. Neurosci. 9:414. doi: 10.3389/fnhum.2015.00414, PMID: 26283943 PMC4516876

[ref24] Latreille V. Ellenrieder N. Peter-Derex L. Dubeau F. Gotman J. Frauscher B. (2020). The human K-complex: insights from combined scalp-intracranial EEG recordings. Neuro Image 213:116748. doi: 10.1016/j.neuroimage.2020.116748, PMID: 32194281

[ref25] Li Y. Dong X. (2023). A RUSBoosted trees method for k-complexes detection using tunable-Q factor wavelet transform and multi-domain feature extraction. Front. Neurosci. 17, 1–14. doi: 10.3389/fnins.2023.1108059, PMID: 36998730 PMC10043251

[ref26] Li Y. Xie S. Zhao J. Liu C. Xie X. (2017). Improved GP algorithm for the analysis of sleep stages based on grey model. Sci. Asia 43, 312–318. doi: 10.2306/scienceasia1513-1874.2017.43.312

[ref27] Migotina D. Rosa A. Fred A. (2010). Automatic k-complex detection using Hjorth parameters and fuzzy decision. Proceedings of the 2010 ACM symposium on applied computing. (Sierre Switzerland).

[ref28] Nawaz R. Cheah K. H. Nisar H. Yap V. V. (2020). Comparison of different feature extraction methods for EEG-based emotion recognition. Biocybern. Biomed. Eng. 40, 910–926. doi: 10.1016/j.bbe.2020.04.005

[ref29] Oliveira G. H. B. S. Coutinho L. R. Silva J. C. D. Pinto I. J. P. Ferreira J. M. S. Silva F. J. S.. (2020). Multitaper-based method for automatic k-complex detection in human sleep EEG. Expert Syst. Appl. 151, 113331–113316. doi: 10.1016/j.eswa.2020.113331

[ref30] Parekh A. Selesnick W. Rapoport I. M. Ayappa I. (2015). Detection of k-complexes and sleep spindles (DETOKS) using sparse optimization. J. Neurosci. Methods 251, 37–46. doi: 10.1016/j.jneumeth.2015.04.006, PMID: 25956566

[ref31] Peker M. (2016). An efficient sleep scoring system based on EEG signal using complex-valued machine learning algorithms. Neurocomputing 207, 165–177. doi: 10.1016/j.neucom.2016.04.049

[ref32] Ranjan R. Arya R. Fernandes S. L. Sravya E. Jain V. (2018). A fuzzy neural network approach for automatic k-complex detection in sleep EEG signal. Pattern Recogn. Lett. 115, 74–83. doi: 10.1016/j.patrec.2018.01.001

[ref33] Shi M. Yang C. Zhang D. (2021). A smart detection method of sleep quality using EEG signal and long short-term memory model. Math. Probl. Eng. 2021, 1–8. doi: 10.1155/2021/5515100

[ref34] Tokhmpash A. Hadipour S. Shafai B. (2021). Epileptic seizure detection using tunable Q-factor wavelet transform and machine learning. Adv. Neuroergon. Cognitive Eng. 259, 78–85. doi: 10.1007/978-3-030-80285-1_10

[ref35] Vu H. Q. Li G. Sukhorukova N. S. Beliakov G. Philippe C. Amiel H.. (2012). K-complex detection using a hybrid-synergic machine learning method. IEEE Trans. Syst. Man Cybern. Part C Appl. Rev. 42, 1478–1490. doi: 10.1109/TSMCC.2012.2191775

[ref36] Yang X. Che H. Leung M.-F. Liu C. (2022). Adaptive graph nonnegative matrix factorization with the self-paced regularization. Appl. Intell. 53, 15818–15835. doi: 10.1007/s10489-022-04339-w

[ref37] Yang M.-S. Nataliani Y. (2018). A feature-reduction fuzzy clustering algorithm based on feature-weighted entropy. IEEE Trans. Fuzzy Syst. 26, 817–835. doi: 10.1109/TFUZZ.2017.2692203

[ref38] Yücelbaş C. Yücelbaş Ş. Özşen S. Tezel G. Küççüktürk S. Yosunkaya Ş. (2018). A novel system for automatic detection of k-complexes in sleep EEG. Neural Comput. & Applic. 29, 137–157. doi: 10.1007/s00521-017-2865-3

[ref39] Zacharaki E. I. Pippa E. Koupparis A. Kokkinos V. Kostopoulos G. K. Megalooikonomou V. (2013). "One-class classification of temporal EEG patterns for k-complex extraction", in: 35th annual international conference of the IEEE EMBS. (Osaka, Japan: IEEE). 10.1109/EMBC.2013.6610870 24111057

[ref40] Zhang X. Landsness E. C. Chen W. Miao H. Tang M. Brier L. M.. (2022). Automated sleep state classification of wide-field calcium imaging data via multiplex visibility graphs and deep learning. J. Neurosci. Methods 366:109421. doi: 10.1016/j.jneumeth.2021.109421, PMID: 34822945 PMC9006179

[ref41] Zhao Z. Liu H. (2007). Searching for interacting features. Proceedings of the 20th international joint conference on artificial intelligence. (Hyderabad, India: Morgan Kaufmann publishers Inc., 340 pine street, sixth floor San Francisco, CA, United States).

[ref42] Zorick T. Smith J. (2016). Generalized information equilibrium approaches to EEG sleep stage discrimination. Comput. Math. Methods Med. 2016, 1–8. doi: 10.1155/2016/6450126, PMID: 27516806 PMC4969566

